# Management of calcified right atrial myxoma extending into the inferior vena cava: a case report

**DOI:** 10.1093/jscr/rjad568

**Published:** 2023-10-17

**Authors:** Mohammad Qaiser Aziz Khan, Amna Zaheer, Sarib Bin Yasir, Ramsha Fatima, Ayush Anand

**Affiliations:** Liaquat National Hospital and Medical College, Karachi 75300, Pakistan; Liaquat National Hospital and Medical College, Karachi 75300, Pakistan; Liaquat National Hospital and Medical College, Karachi 75300, Pakistan; Liaquat National Hospital and Medical College, Karachi 75300, Pakistan; B. P. Koirala Institute of Health Sciences, Dharan 56700, Nepal

**Keywords:** atriotomy, cardiac myxoma, case report, right atrial myxoma

## Abstract

Cardiac myxomas can rarely involve the right atrium, get calcified and involve the inferior vena cava (IVC). Early surgical intervention is critical to prevent life-threatening complications. We presented the case of a 39-year-old male with fever, cough and shortness of breath for 1 week. Initial laboratory investigations revealed leucocytosis and thrombocytopenia. His electrocardiogram was normal, and his chest X-ray showed bilateral infiltrates. Based on the findings of a high-resolution chest computed tomography scan, echocardiography and cardiac magnetic resonance imaging, we made a provisional diagnosis of calcified right atrial myxoma extending up to the IVC. We managed the case with cardiopulmonary bypass via aortic cannulation followed by a right atriotomy. Post-operatively, the patient’s condition improved and was doing well on monthly follow-ups.

## Introduction

Primary cardiac tumours are rarely reported, with an incidence ranging from 0.001 to 0.30% [1]. Cardiac myxomas account for approximately half of all adult primary cardiac tumours [2, 3]. Most commonly, cardiac myxomas arise from the left atrium [4]. Rarely, it can arise from the right atrium involving the inferior vena cava (IVC) [4, 5]. The patient’s presentation age can vary from 30 to 60 years, with the mean age of diagnosis at 50 [1]. Most commonly, the patients present with dyspnoea and constitutional symptoms [1, 6]. Usually, these patients are diagnosed incidentally following evaluation of the cardiac lesion [1]. A computed tomography scan of the chest, transthoracic echocardiography and cardiac magnetic resonance imaging (MRI) can be used to evaluate cardiac lesions and rule out differentials [3, 7]. The mainstay of management is early surgical resection of the tumour to prevent life-threatening complications [7, 8].

## Case report

A 39-year-old male complained of fever, cough and shortness of breath for one week. His past history, medical history and personal history were unremarkable. On examination, his vitals were blood pressure of 110/70 mm of Hg, pulse rate of 100 beats per minute, respiratory rate of 26 breaths/min, temperature of 100.5 °F and 97% oxygen saturation on room air. Physical examination reveals bilateral crackles, more on the left chest. The cardiovascular examination did not reveal any abnormalities. The rest of the systemic examinations were unremarkable.

**Figure 1:**
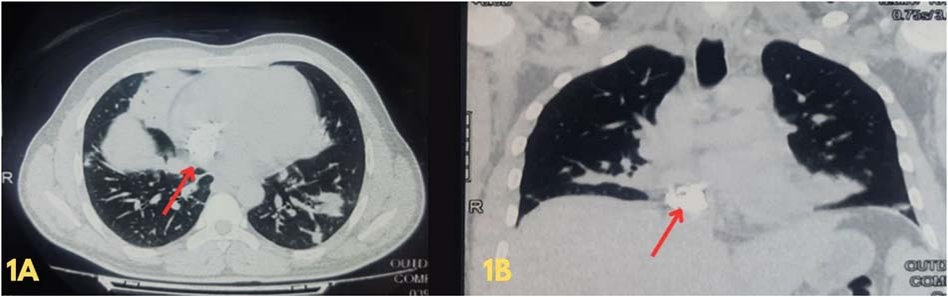
(A and B) Axial and coronal views of a high-resolution computed tomography scan of the chest showing multiple opacifications in the lung field and heavy calcification within the right atrium, extending up to the opening of the inferior vena cava.

**Figure 2:**
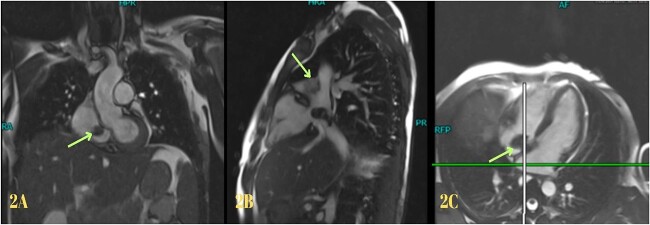
Cardiac magnetic resonance imaging showing myxoma.

Blood investigations revealed leukocytosis and thrombocytopenia (Table 1). His electrocardiogram (ECG) showed a normal sinus rhythm, and chest X-ray revealed bilateral infiltrates. A high-resolution computed tomography (HRCT) scan of the chest was done in February 2020, which showed multiple patchy pulmonary infiltrates in the upper and lower lobe of the left lung, the middle lobe and the lower lobe of the right lung (Fig. 1). An air bronchogram was noted within the area of consolidation of the middle lobe of the right lung. Also, heavy calcification of irregular areas within the right atrium, extending up to the opening of the IVC, was noted. We did a further investigation with transthoracic echocardiography, which revealed an echogenic mass in the right atrium of 3.0 ✕ 1.9 cm^2,^ most likely a thrombus. Following this, we investigated the patient with cardiac MRI, which showed a large irregular mass of 25 ✕ 18 mm^2^ in the right atrium attached to the interatrial septum and extending to the IVC, most likely myxoma with superimposed thrombus (Fig. 2).

**Table 1:** Initial laboratory investigations of the patient.

**Investigations**	**Results**	**Reference range**
Hb (g/dl)	11.89	13.5–17.50 g/dl
TLC (cells/mm^3^)	14 430	40 000–11 000
Platelet count (cells/mm^3^)	54 000	150 000-400 000
Serum urea (mg/dl)	28	<50 mg/dl
Serum creatinine (mg/dl)	1.03	0.5–1.5 mg/dl
Serum potassium (mmol/l)	4.7	3.5–5.3
Serum sodium (mmol/l)	144	136–145
ALT (U/l)	67	<41 U/L
AST(U/l)	58	<50 U/L
ALP (U/l)	216	<129 U/L
Blood urea nitrogen (mg/dl)	19	8–24

Hb: haemoglobin; TLC: total leucocyte count; ALT: alanine transaminase; AST: aspartate transaminase; ALP: alkaline phosphatase.

**Figure 3:**
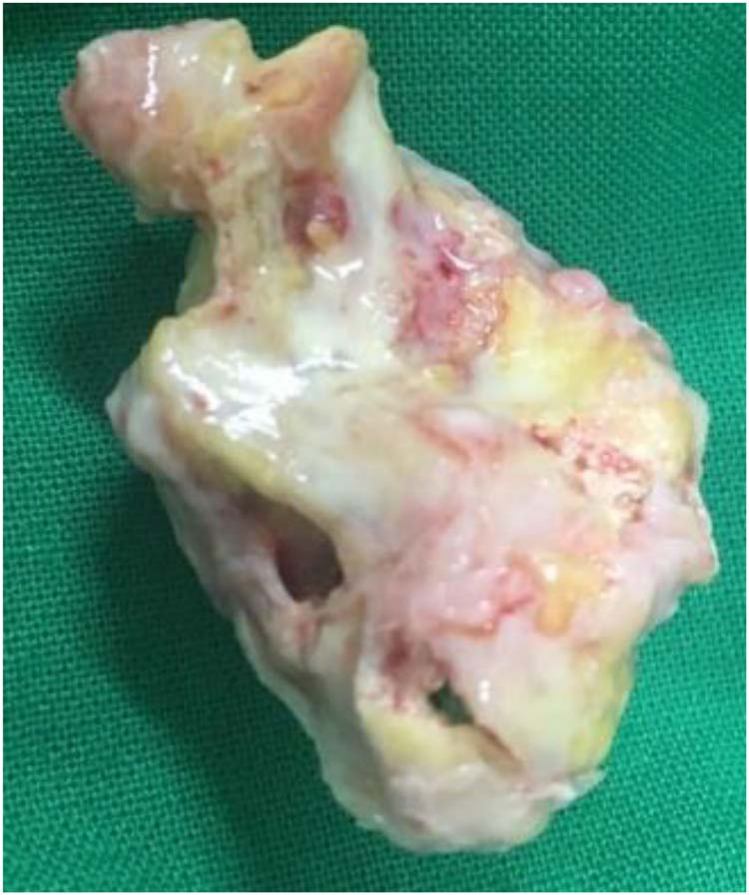
Gross morphology of the resected specimen.

**Figure 4:**
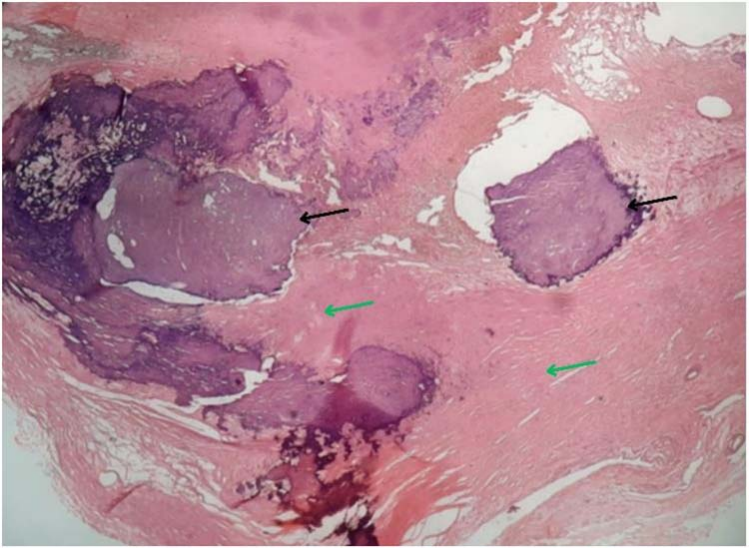
Microscopy of the resected specimen showing focal calcified (black arrow) and myxoid areas.

Based on the clinical evaluation, we made a provisional diagnosis of calcified right atrial myxoma extending up to the IVC. We planned for surgical resection of the tumour. We cannulated the right femoral vein and superior vena cava. Then, we did a cardiopulmonary bypass via aortic cannulation followed by a right atriotomy. Standard cardiopulmonary bypass was used during the procedure. The IVC was cannulated with extreme care. We kept the patient in the Trendelenburg position with a venous cannula (32Fr) to ease the introduction of the bulk away from the IVC orifice and away from the cannula. To make the mass easier to remove and prevent fragmentation, entrapping of the IVC was avoided. To better expose the IVC opening, we underwent a right atriotomy in the shape of a T. A solid, spherical, reddish-white lump of 1.8 × 1.8 cm^2^ in size was found with a broad base linked to the right atrium’s anterior superior IVC junction. The entire mass was removed, and its base was cauterized. The mass was hard, calcified, attached to the right atrium, and extended into IVC. A gross morphology examination revealed a single irregular greyish-white piece of tissue measuring (4.2 × 3.3 × 2.5) cm^3^ and weighing 15 g (Fig. 3). Microscopy revealed multiple fragments of extensively calcified fibrous lesions with focally abundant myxoid stroma with stellate cells, necrosis mitotic activity, atypia and pleomorphism were absent, which confirmed the diagnosis of calcified atrial myxoma (Fig. 4). The postoperative course was unremarkable, and the patient reported symptomatic improvement in the follow-up visit after one month of operation. During the postoperative follow-up, it was noted that the patient had normal vitals and no signs of infection. The preoperative symptoms of shortness of breath and chest heaviness had been entirely resolved. The patient was able to resume daily activities without any difficulty. The ECG and other cardiac tests revealed no abnormal findings, indicating the patient’s heart function had been restored to normal.

## Discussion

Cardiac myxoma involving the right atrium and extending into the IVC with calcification is rarely reported in the literature [4, 5]. Cardiac myxomas can sometimes be familial and associated with the carney complex [1]. Usually, females are more affected, and the majority of patients are in the third to ninth decade of life [1]. Our patient was a male in his thirties, and we did not report any feature suggestive of an associated carney complex. The clinical presentation can range from asymptomatic to symptomatic depending upon the obstruction of the blood flow in cardiac chambers and pulmonary circulation and interference with valvular function [1, 3]. Patients can also have constitutional symptoms [1]. Also, laboratory findings such as anaemia, leukocytosis, increased sedimentation and thrombocytopenia can be reported in these patients [1, 7, 9]. Our patient had a fever, cough and dyspnoea, suggesting pulmonary or cardiac causes. Similar to other cases, the blood test results revealed thrombocytopenia and leukocytosis in our patient [1, 3, 7, 9].

Usually, these tumours are diagnosed incidentally while evaluating the cardiac lesion in a patient [1]. Diagnosis can be made based on clinical, imaging and histopathology findings [3, 7]. Imaging modalities such as HRCT, echocardiography and cardiac MRI can aid in diagnosis [1, 3, 7]. Typically, histopathology can reveal polygonal, spindle and stellate cells in a myxomatous background [10]. Radiological investigations such as chest x-ray, HRCT, and cardiac MRI were used to rule out differentials suggesting cardiac myxoma. In addition, the histopathology of the resected specimen also suggested cardiac myxoma. Hence, we made a diagnosis of cardiac myxoma. Early diagnosis and intervention of cardiac myxomas are associated with a good prognosis [7, 8]. Surgical intervention is the mainstay of management for cardiac myxoma [1]. Hence, we managed the patient with complete tumour resection, and the patient improved symptomatically.

## Data Availability

All relevant data pertaining to this case is made available within the manuscript.
